# Arterial calcification on preoperative computed tomography imaging as a risk factor for pharyngocutaneous fistula formation after total laryngectomy

**DOI:** 10.1002/hed.26927

**Published:** 2021-11-10

**Authors:** Sandra I. Bril, Najiba Chargi, Thomas F. Pezier, Bernard M. Tijink, Weibel W. Braunius, Ernst J. Smid, Pim A. de Jong, Remco de Bree

**Affiliations:** ^1^ Department of Head and Neck Surgical Oncology University Medical Center Utrecht, Utrecht University Utrecht The Netherlands; ^2^ Department of Radiation Oncology University Medical Center Utrecht, Utrecht University Utrecht The Netherlands; ^3^ Department of Radiology and Nuclear Medicine University Medical Center Utrecht, Utrecht University Utrecht The Netherlands

**Keywords:** computed tomography, head and neck cancer, laryngectomy, postoperative complications, vascular calcification

## Abstract

**Background:**

Research in esophageal surgery showed that computed tomography (CT) assessed arterial calcification (AC) is associated with postoperative complications. We investigated the association between AC and pharyngocutaneous fistula (PCF) formation after laryngectomy.

**Methods:**

This was a retrospective cohort study of patients undergoing laryngectomy. AC was scored at 10 different anatomical locations on CT imaging, blinded for PCF occurrence. Association with PCF was investigated using logistic regression.

**Results:**

The 224 patients were included; 62 (27.7%) developed a PCF. Moderate to severe AC was widespread in patients undergoing TL; 7.1% of patients had at most mild AC, of whom 1 experienced a PCF (*p* = 0.05). A higher cumulative calcification score was associated with PCF in univariable (OR 1.11, *p* = 0.04) and multivariable analysis (OR 1.14, *p* = 0.05).

**Conclusion:**

AC is widespread in patients undergoing laryngectomy and its burden is associated with PCF. Extensive AC on preoperative imaging may be considered a risk factor for PCF.

## 1 INTRODUCTION

Total laryngectomy (TL) is a definitive treatment for patients with advanced stage laryngeal or pharyngeal cancer. It is also a salvage treatment option for patients with recurrent disease after (chemo)radiotherapy, and can be used to treat a dysfunctional larynx.^1^, ^2^ It is an invasive surgical procedure and associated with frequent postoperative complications resulting in significant morbidity and mortality, compromising survival and quality of life.^3^, ^4^, ^5^

Postoperative complications, including wound healing problems and the occurrence of a pharyngocutaneous fistula (PCF), are common and difficult to treat. Approximately 30% of patients develops a PCF after TL, which often requires additional surgery, flap reconstruction, prolonged hospital stay and feeding tube dependency.^6^, ^7^ Known risk factors for PCF are prior chemoradiotherapy, hypopharyngeal cancer, extensive pharyngeal resection and reconstruction, neck dissection, and low body mass index (BMI). Recently, radiologically assessed sarcopenia or low skeletal muscle mass has been identified as a novel risk factor for PCF in patients undergoing TL.^8^, ^9^

Research shows that routinely performed imaging, such as computed tomography (CT), can be used to extract additional information on patient's body composition as a biomarker of functional and biological status, next to cancer specific features and risk factors.^10^, ^11^, ^12^ The radiological assessment of sarcopenia is an example of this. Routinely performed CT imaging can also be used to assess arterial calcification, as evidence of the presence of atherosclerosis.^13^, ^14^ In head and neck cancer patients, head and neck CT imaging is commonly performed during the diagnostic work‐up, on which the carotid arteries and vertebral arteries are shown. Additionally, thoracic CT imaging and/or whole body FDG‐PET/CT imaging may be performed, depending on local diagnostic protocols, which provides additional imaging of the heart and aorta. Smoking, a known etiological factor for atherosclerosis, is common in head and neck cancer patients.^15^ Another etiological factor for atherosclerosis is a low‐level persistent systemic inflammation, which is also commonly observed in cancer patients.^16^, ^17^ It may be hypothesized that atherosclerosis is a risk factor for wound healing in surgery due to inadequate perfusion at the site of surgery and microvascular dysfunction. Recent studies in patients with esophageal cancer undergoing esophagectomy showed that locoregional and generalized cardiovascular disease as identified on routine CT imaging was predictive of cervical anastomotic leakage.^18^, ^19^ Another study in patients with rectal cancer showed that calcification of the iliac arteries was associated with anastomotic leakage after colorectal surgery with rectal anastomosis.^20^

The purpose of this study was to explore the extent of arterial calcifications present in patients undergoing TL and to investigate whether the presence and burden of regional and generalized atherosclerotic calcification, as visualized on preoperative CT imaging, is a risk factor for PCF in patients undergoing TL.

## 2 PATIENTS AND METHODS

This study is a retrospective cohort study on which we previously reported.^8^ The design of this study was approved by the Medical Ethical Research Committee of the University Medical Center Utrecht (ID 17‐365/C). The research was conducted in accordance with the Declaration of Helsinki.

### 2.1 Patient and study design

All patients who had undergone TL between January 2008 and May 2017 at our institution were considered for inclusion. Patients were discussed in the local tumor board meeting, and all patients who were included underwent TL with or without (partial) pharyngectomy and with or without additional lymph node dissection; either as primary treatment, as salvage treatment for recurrent or residual tumor after prior (chemo)radiotherapy treatment, or as functional treatment for a dysfunctional larynx after prior (chemo)radiotherapy, where no vital tumor was found. Five dedicated head and neck surgical oncologists performed all TL. Exclusion criteria for this analysis included insufficient quality CT imaging as determined by an experienced radiologist or the absence of CT imaging (e.g., only MRI imaging performed).

Patients' demographic, tumor stage, treatment, and outcome data were collected using electronic patient records. Operating records were checked for details of the surgery, neck dissection, and primary pharyngeal closure or flap reconstruction of the pharynx. The occurrence of PCF was defined as a clinical fistula requiring conservative or surgical treatment. In patients who had surgery for a dysfunctional larynx, the tumor site for which the patient received prior treatment was documented. Follow‐up data were retrieved up until August 31, 2017.

The presence of sarcopenia was assessed on preoperative CT imaging using a previously specified protocol. In brief, the cross‐sectional skeletal muscle area at the level of C3 was measured on a single transversal CT slice at the level of the third cervical vertebra (C3).^8^, ^12^ The cross‐sectional muscle area was normalized for height to calculate the skeletal muscle index. A skeletal muscle index of below 43.2 cm^2^/m^2^ was deemed to be sarcopenia.^21^

### 2.2 Image acquisition

All CT imaging was routinely performed at our hospital. Patients underwent contrast‐enhanced CT scanning of the head and neck area on a Philips scanner with 64 detector rows or more (Philips Healthcare, Best, The Netherlands) at our institution. All routine diagnostic CT protocols include thin slices (<1 mm) and reconstruction at 3–5 mm.

### 2.3 Image evaluation

Images were typically analyzed in multiple directions by one reader (P.A.d.J), a radiologist with >10 years of experience in CT evaluation and a specific research interest in arterial calcification. The reader was blinded for patient and treatment related factors, as well as for study outcomes (e.g., formation of PCF).

**FIGURE 1:**
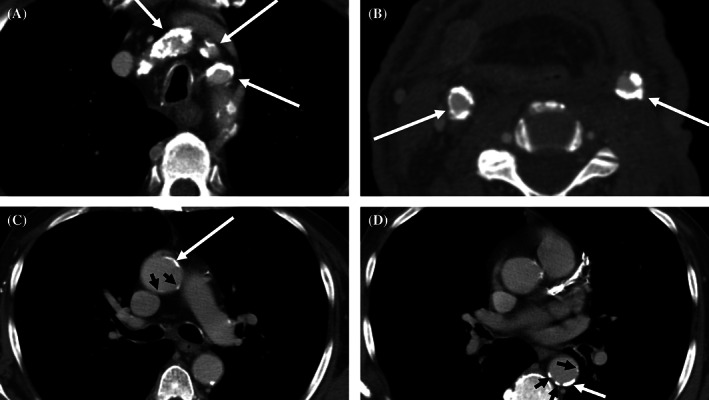
Examples of preoperative CT images of arterial calcification in patients planned for laryngectomy. A white arrow indicates a moderate to severe calcification (score 2 or 3) whereas a black arrow indicates minor calcification (score 1). (A) Calcification of the origins of all three brachiocephalic arteries, resulting in a score of 3 for calcification of the origins of the brachiocephalic arteries. (B) Calcification of the left and right external carotid arteries, resulting in a score of 3 for calcification of the external carotid arteries. (C) Calcification of the ascending aorta with two minor specs of calcification and one severe calcification, resulting in a score of 2 for calcification of the ascending aorta. (D) Multiple calcified foci of the descending aorta with several minor specs of calcification and one larger segment, resulting in a score of 2 for calcification of the descending aorta

A visual grading system was used similar to previous studies in order to consistently score CT images on arterial calcification at 10 different anatomical locations.^18^, ^22^ The selected locations include large aortic structures (ascending aorta, aortic arch, descending aorta and origins of the brachiocephalic arteries), carotid structures (left and right extracranial carotid artery, left and right carotid siphon), and left and right vertebral arteries. Scores of 0, 1, 2, and 3 were assigned, for all locations except the origins of the brachiocephalic arteries corresponding with the absence of calcifications (score 0), mild calcification defined as one or two dots of calcium smaller than 1 cm (score 1), moderate calcification defined as one calcification larger than 1 cm (score 2), and severe calcification defined as circular calcification or a large calcification combined with smaller dots or >2 dots (score 3), respectively. For the origins of the brachiocephalic arteries, a score of 0 corresponds with no calcification present, a score of 1 with the calcification of one origin of a brachiocephalic artery, a score of 2 with the calcification of two brachiocephalic arteries and a score of 3 with the calcification of all three brachiocephalic arteries. A cumulative calcification score was calculated of arterial calcification scores at all anatomical sites resulting in a score between 0 and 30 for total arterial calcification. Table 1 shows the distribution of arterial calcification at the selected anatomical locations. Examples of arterial calcification on CT imaging are presented in Figure **1**.

**TABLE 1:** Distribution of arterial calcification on preoperative CT images

Anatomical location	Calcification scores n (% of total)				Missing n (% of total)
	0—Absent	1—Mild	2—Moderate	3—Severe	
Ascending aorta	78 (34.8)	49 (21.9)	20 (8.9)	30 (13.4)	47 (21.0)
Aortic arch	37 (16.5)	28 (12.5)	56 (25.0)	78 (34.8)	25 (11.2)
Descending aorta	43 (19.2)	27 (12.1)	27 (12.1)	77 (34.4)	50 (22.3)
Origins of the brachiocephalic arteries	31 (13.8)	30 (13.4)	36 (16.1)	117 (52.2)	10 (4.5)
Left extracranial carotid artery	37 (16.5)	24 (10.7)	46 (20.5)	116 (51.8)	1 (0.4)
Right extracranial carotid artery	39 (17.4)	30 (13.4)	45 (20.1)	109 (48.7)	1 (0.4)
Left vertebral artery	176 (78.6)	18 (8.0)	13 (5.8)	13 (5.8)	4 (1.8)
Right vertebral artery	181 (80.8)	17 (7.6)	13 (5.8)	9 (4.0)	4 (1.8)
Left carotid siphon	33 (14.7)	31 (13.8)	46 (20.5)	104 (46.4)	10 (4.5)
Right carotid siphon	35 (15.6)	32 (14.3)	40 (17.9)	107 (47.6)	10 (4.5)

### 2.4 Statistical analysis

Categorical data are represented as a number and percentage of the total. A test for normality (Kolmogorov–Smirnoff test) was used to assess whether continuous variables were normally distributed. Continuous data are represented as mean ± SD if normally distributed, and median ± interquartile range if skewed. Fisher's exact tests, Pearson Chi square tests, independent sample *t* tests, and Mann–Whitney *U* tests were used to assess group differences where appropriate. Univariable logistic regression analysis and multivariable logistic regression analysis using backward stepwise selection was used to examine the association between calcifications and PCF. Covariates in regression analysis were chosen based association with PCF in univariable logistic regression analysis (*p* <0.05). If there was a significant association between two variables, the variable with the strongest association with PCF was entered in multivariable logistic regression analysis.

Not all anatomical locations could be assessed in all patients, most commonly when CT imaging of the thoracic area was not available or because of dental artifacts. The missing data were considered missing at random. Multiple imputation of these missing sites was applied to replace the missing values for logistic regression analysis, using the median of 20 imputated datasets.^23^, ^24^

Statistical analyses was performed using the IBM SPSS Statistics version 25.0 software package (Chicago, IL, USA). All analyses were two‐sided and *p* ≤0.05 was considered significant.

## 3 RESULTS

Between January 2008 and June 2017, 245 patients underwent TL at our institution. Of these 245, 17 patients were excluded because there was no CT imaging available, and 4 patients were excluded because of inadequate quality of imaging. Therefore, 224 patients were included in this analysis. Median interval between imaging and TL was 27 days.

### 3.1 Patient and treatment characteristics

The 224 patients that were included for analysis had a mean age of 64.8 years. Patients were predominantly male (82.1%). During the study period, 105 patients (46.9%) underwent primary TL, 108 patients (48.2%) underwent salvage TL, and 11 patients (4.9%) underwent a functional TL. Prior to TL, 98 patients (43.8%) had undergone radiotherapy and 21 patients (9.4%) had undergone chemoradiotherapy. Of the patients undergoing functional TL, 1 had undergone prior chemoradiotherapy, and 10 had undergone prior radiotherapy.

A PCF occurred in 62 patients (27.7%), which required surgical closure in 40 patients (64.5% of all PCF). Patient, disease, and treatment related characteristics, and their relationship with the occurrence of a PCF are presented in Table **2**.To summarize, patients who had a PCF more often had hypopharyngeal cancer, a dysfunctional larynx after treatment, sarcopenia, laryngectomy with pharyngectomy and flap closure of the neopharynx. Additional lymph node dissection and prior treatment for head and neck cancer not resulting in laryngeal dysfunction was not more common in patients with a PCF. Patients with a PCF were not significantly older and did not have a significantly lower BMI.

**TABLE 2:** Patient, disease and treatment characteristics

Characteristic	With pharyngocutaneous fistula n = 62 (% of total)	Without pharyngocutaneous fistula n = 162 (% of total)	*p* value
Sex (male)	48 (77.4)	136 (84.0)	0.25^a^
Age at diagnosis in years (SD)	64.0 (9.2)	65.1 (9.1)	0.43^b^
Body mass index (SD)	23.4 (4.8)	24.2 (5.1)	0.32^b^
Smoking (current)	30 (48.4)	82 (50.6)	0.33^a^
Alcohol abuse^c^			0.44^d^
Never	37 (59.7)	111 (68.5)	
Former	17 (27.4)	33 (20.4)	
Current	8 (12.9)	18 (11.1)	
ASA classification			0.84^d^
I	28 (45.2)	80 (49.4)	
II	18 (29.0)	42 (25.9)	
III	16 (25.8)	40 (24.7)	
Presence of sarcopenia			**0.04** ^a^
No	27 (43.5)	95 (58.6)	
Yes	35 (56.5)	67 (41.4)	
Localization tumor			**<0.01** ^a^
Larynx	34 (54.8)	132 (81.5)	
Hypopharynx	28 (45.2)	30 (18.5)	
AJCC stage			**0.01** ^d^
0	7 (11.3)	4 (2.5)	
I	3 (4.8)	22 (13.6)	
II	11 (17.7)	21 (13.0)	
III	6 (9.7)	28 (17.3)	
IV	35 (56.5)	87 (53.7)	
Indication for TL			
Primary HNC	26 (41.9)	79 (48.8)	**0.02** ^d^
Recurrent HNC	29 (46.7)	79 (48.8)	
Dysfunctional larynx	7 (11.3)	4 (2.5)	
Prior treatment			
None	26 (41.9)	79 (48.8)	0.62^d^
Radiotherapy	29 (46.8)	69 (42.6)	
Chemo‐radiotherapy	7 (11.3)	14 (8.6)	
Type resection			**<0.01** ^a^
Laryngectomy	31 (50.0)	120 (74.1)	
LE + pharyngectomy	31 (50.0)	42 (25.9)	
Closure of neopharynx			**0.03** ^a^
Direct	33 (53.2)	123 (75.9)	
Flap closure	29 (46.8)	39 (24.1)	
Lymph node dissection			0.23^a^
No	22 (35.5)	73 (45.1)	
Yes	42 (67.7)	89 (54.9)	

*Note*: The significant different values are bold.

a Fisher's exact test.

b Independent sample *t* test.

c Defined as the consumption of 5 of more units per day for men and 4 or more units per day for women; or a medical history note including alcohol abuse.

d Pearson Chi squared test.

### 3.2 Arterial calcification and univariable analysis

Distribution of arterial calcifications is shown in Table 1. In the majority of patients, presence of arterial calcifications was found. Arterial calcifications of the aortic artery and carotid branches were a common finding. In contrast, arterial calcifications in the vertebral arteries were rare. Only 3 patients (1.3%) had no arterial calcification. In 16 patients (7.1% of total), at most mild calcifications were seen. Of those 16 patients, only one patient had a PCF (Pearson Chi square test: *p* = 0.05). For subsequent analysis calcification scores were divided in two groups; none to mild calcifications, and moderate to severe calcifications.

Distribution of arterial calcification among patients with and without PCF and univariable odds ratios are shown in Table **3**. Arterial calcifications in all anatomical locations apart from the vertebral arteries were more frequently observed in patients who had a PCF, which is shown in Table **3**. Arterial calcification of the descending aorta (OR 2.32, 95% CI 1.25–4.29, *p* = 0.01), origins of the brachiocephalic arteries (OR 2.14, 95% CI 1.05–4.35, *p* = 0.04), right extracranial carotid artery (OR 2.05, 95% CI 1.03–4.09, *p* = 0.04) and left carotid siphon (OR 2.26, 95% CI 1.12–4.59, *p* = 0.02) were significantly associated with PCF formation. A higher total arterial calcification score was significantly associated with PCF formation (OR 1.11, 95% CI 1.00–1.27, *p* = 0.04). When the vertebral arteries were left out of the arterial calcification score, the association with PCF was stronger: adapted arterial calcification score OR 1.19, 95% CI 1.04–1.36, *p* = 0.01.

**TABLE 3:** Distribution of arterial calcification in patients with and without pharyngocutaneous fistula and univariate odds ratio

Anatomical location of arterial calcification	Score^a^	With pharyngocutaneous fistula (n = 62; % of total)	Without pharyngocutaneous fistula (n = 162; % of total)	Unadjusted OR^b^ (95% CI)	*p* value
Ascending aorta	0	31 (50.0)	101 (63.2)	1.66 (0.92–2.90)	0.09
	1	31 (50.0)	61 (37.7)		
Aortic arch	0	20 (32.3)	56 (34.6)	1.11 (0.60–2.07)	0.74
	1	42 (67.7)	106 (65.4)		
Descending aorta	0	20 (32.3)	85 (52.5)	2.32 (1.25–4.29)	**0.01**
	1	42 (67.7)	77 (47.5)		
Origins of the brachiocephalic arteries	0	12 (19.4)	55 (34.0)	2.14 (1.05–4.35)	**0.04**
	1	50 (80.6)	107 (66.0)		
Left extracranial carotid artery	0	16 (25.8)	46 (28.4)	1.14 (0.59–2.21)	0.70
	1	46 (74.2)	116 (71.6)		
Right extracranial carotid artery	0	13 (21.0)	57 (35.2)	2.05 (1.03–4.09)	**0.04**
	1	49 (79.0)	105 (64.8)		
Left vertebral artery	0	58 (93.5)	136 (84.0)	0.36 (0.12–1.08)	0.07
	1	4 (6.5)	26 (16.0)		
Right vertebral artery	0	57 (91.9)	141 (87.0)	0.59 (0.21–1.64)	0.31
	1	5 (8.1)	21 (13.0)		
Left carotid siphon	0	12 (19.4)	57 (35.2)	2.26 (1.12–4.59)	**0.02**
	1	50 (80.6)	105 (64.8)		
Right carotid siphon	0	15 (24.2)	60 (37.0)	1.84 (0.95–3.58)	0.07
	1	47 (75.8)	102 (63.0)		
Total arterial calcification score^c^	Median	7.0	6.0	1.11 (1.00–1.27)	**0.04**
	IQR^d^	5.0–8.0	3.0–7.0		
Adapted arterial calcification score^e^	Median	7.0	5.0	1.19 (1.04–1.36)	**0.01**
	IQR^d^	5.0–7.0	3.0–7.0		

*Note*: Numbers in bold: significant at the level of *p* ≤0.05.

^a^ Score: 0—none to mild; 1—moderate to severe.

^b^ Univariable logistic regression analysis.

^c^ Continuous; cumulative arterial calcification score at all 10 anatomical locations. Score between 0 and 10.

^d^ Interquartile range.

^e^ Continuous; cumulative arterial calcification score at aortic and carotid anatomical locations. Score between 0 and 8.

### 3.3 Multivariable logistic regression analysis

The total calcification score (cumulative arterial calcification scores at all 10 anatomical locations) and adapted arterial calcification score (cumulative arterial calcification scores of aortic and carotid structures) were tested in a multivariable logistic regression model as a predictor for fistula formation, together with patient related and operative risk factors for PCF. Variables entered in the multivariable model were included if they were associated with PCF in univariable analysis (*p* <0.05). In univariable analysis, presence of sarcopenia, localization of the tumor, AJCC stage, indication of TL, pharyngeal resection and closure of the neopharynx were significantly associated with the occurrence of a PCF. The results of the univariable logistic regression analysis and multivariable logistic regression analysis is shown in Table **4**. There was a significant interaction between indication for TL and AJCC stage, where patients undergoing TL for functional reasons are scored AJCC stage 0, which was highly associated with PCF, only indication for TL was entered in multivariable analysis. Because of a significant interaction between localization of the tumor and pharyngeal resection, only localization of the tumor was entered in multivariable analysis.

**TABLE 4:** Univariable and multivariable logistic regression analysis for total arterial calcification score and adapted arterial calcification score as a risk factor for pharyngocutaneous fistula

Risk factor	Univariable OR (95% CI)	*p* value	Multivariable OR (95% CI)	*p* value
Presence of sarcopenia				0.08
No	Ref		Ref	
Yes	1.96 (1.09–3.55)	**0.03**	1.76 (0.94–3.28)	
Indication for TL				
Primary HNC	Ref		Ref	0.35
Recurrent HNC	1.01 (0.55–1.87)	0.97	1.37 (0.70–2.67)	
Dysfunctional larynx	5.06 (1.37–18.63)	**0.02**	3.78 (0.95–15.16)	
Localization tumor				0.06
Larynx	Ref		Ref	
Hypopharynx	3.62 (1.91–6.86)	**<0.01**	3.75 (1.91–7.36)	
Closure of neopharynx				**<0.01**
Direct	Ref	**<0.01**	Ref	
Flap	2.77 (1.50–5.13)		1.73 (0.78–3.82)	
Type of resection		**<0.01**		0.18
Laryngectomy	Ref			
LE + pharyngectomy	2.86 (1.55–5.26)			
AJCC stage				
I	Ref			
II	3.84 (0.94–15.73)	**0.06**		
III	1.57 (0.35–7.00)	0.55		
IV	2.95 (0.83–10.49)	0.10		
0 (functional TL)	12.83 (2.29–71.29)	**<0.01**		
Total arterial calcification score^a^	1.11 (1.00–1.27)	**0.04**	1.14 (1.00–1.29)	**0.05**
Adapted arterial calcification score^b^ ^,^ ^c^	1.19 (1.04–1.36)	**0.01**	1.20 (1.04–1.38)	**0.01**

*Note*: Numbers in bold: significant at the level of *p* ≤0.05.

^a^ Continuous; score between 0 and 10.

^b^ Continuous; score between 0 and 8.

^c^ Entered in multivariable logistic regression analysis instead of the total arterial calcification score.

In multivariable analysis, the total arterial calcification score was significantly associated with the occurrence of PCF (OR 1.14, 95% CI 1.00–1.29, *p* <0.05). The adapted arterial calcification score was entered separately in multivariable regression analysis with the other covariates; the adapted arterial calcification score was significantly associated with the occurrence of PCF (OR 1.20, 95% CI 1.04–1.37, *p* = 0.01).).

### 3.4 Arterial calcification and sarcopenia

As there may be a shared etiological factor in atherosclerosis and sarcopenia, the occurrence of arterial calcification in patients with and without sarcopenia was explored. Data are shown in Table S1. Moderate to severe arterial calcification at the location of the descending aorta was significantly more often present in patients with sarcopenia as compared to patients without sarcopenia (Pearson Chi square test: *p* <0.01). At the other locations, no significant difference was observed. The association between arterial calcification and sarcopenia as independent risk factors for PCF formation is shown in Table S2. In multivariable logistic regression analysis, the adapted arterial calcification score (OR 1.18, 95% CI 1.03–1.35, *p* = 0.02) (adjusted OR 1.12, 95% CI 1.00–1.10, *p* = 0.04) and sarcopenia (adjusted OR 1.86, 95% CI 1.02–3.39, *p* = 0.04) were independently associated with PCF formation. There was no significant association between the total arterial calcification score and PCF in multivariable analysis (OR 1.12, 95% CI 1.00–1.26, *p* = 0.06), although a trend may be observed.

## 4 DISCUSSION

This retrospective cohort study of patients undergoing laryngectomy shows that arterial calcification is widespread in patients undergoing laryngectomy, and is associated with PCF formation. Moderate to severe arterial calcifications of the descending aorta, origins of the brachiocephalic arteries and left carotid siphon were significantly associated with developing a PCF. A higher cumulative total arterial calcification score (range 0–10) was significantly associated with the occurrence of PCF: the relative risk of PCF increased by 11% per point increase in total arterial calcification score.

Our results are concurrent with recent studies in esophageal and colorectal surgery. Recent studies in patients undergoing esophagectomy showed that locoregional and generalized cardiovascular disease as identified by visual grading on preoperative imaging was a risk factor for wound healing problems and anastomotic leakage.^18^, ^19^ Another study in patients undergoing colorectal surgery showed that visually graded calcification of the abdominal aorta was associated with increased morbidity after surgery.^25^ It is hypothesized that both locoregional and generalized arterial vascular disease may have a detrimental effect on wound and anastomosis healing due to low flow or hypoperfusion of the surgical area, leading to ischemia.^19^, ^26^

The occurrence of a PCF after TL is a severe complication. It is associated with prolonged hospital stay and feeding tube dependency, as well as decreased quality of life, and it negatively affects survival. Recently, radiologically assessed sarcopenia was identified as a preoperative risk factor for PCF and wound complications in head and neck cancer patients.^8^, ^9^, ^27^ There may be a link between the presence of arterial calcifications and sarcopenia, as systemic inflammation may be a shared etiological factor. The copresence of sarcopenia and arterial calcification was often observed. In multivariable regression analysis, the presence of sarcopenia and arterial calcifications appeared to be both predictors of PCF.

Routinely performed CT imaging provides additional information on patients' functional and biological status, and may aid in the identification of high‐risk patients for the occurrence of adverse outcomes. Accurate identification of high‐risk patients for PCF may provide an opportunity for preoperative interventions to decrease the risk. It seems impossible to decrease the amount of arterial calcifications in the preoperative period, but preoperative optimization of general cardiovascular status or other risk factors associated with PCF, which co‐exist might decrease the risk of a PCF.^28^, ^29^, ^30^ Arterial calcifications as evidence for cardiovascular disease may warrant further examination and intervention prior to surgery. A surgical solution to decrease the risk of PCF in high‐risk patients may be to use a pectoralis major overlay flap to reinforce the suture line of the neopharynx by covering it with healthy muscle.^31^ In reconstructive microsurgery, radiological evidence of atherosclerosis may also aid in choosing the optimal flap for reconstruction.^32^

There are several limitations that need to be addressed. Relevant clinical data such as known cardiovascular disease and diabetes was missing in our database due to missing information in particular in the earlier years of the study period. Also, some traditional cardiovascular risk factors such as serum cholesterol are missing, because these are not routinely measured at our clinic. Smoking and age was included in analysis, and the ASA classification was used as a surrogate for comorbidities, but we acknowledge that this provides limited information on specific comorbidities.^33^ Recent studies suggest that coronary arterial calcification scores or peripheral arterial calcification scores derived from CT imaging are reliable assessment methods for cardiovascular disease, and can identify patients at high risk that would not have been identified using traditional cardiovascular risk factors.^14^, ^34^, ^35^ Second, a visual grading system for arterial calcification as opposed to calcium scores may lead to an observer bias and necessitates a learning curve. Automatic calcium scoring systems are not available using head and neck contrast enhanced CT imaging, but research into automatic arterial calcification scoring on contrast‐enhanced CT imaging is ongoing, and this may in the future be available.^36^, ^37^ Machine learning and radiomics using CT features, for example, skeletal muscle mass, skeletal muscle quality and arterial calcification, from routinely performed head and neck CT imaging may aid in identifying patients at high risk for fistula formation after laryngectomy in the future. In this study, all calcification scoring was performed by one observer; an experienced radiologist with a research interest in and extensive experience with arterial calcification on CT imaging. The inter‐ and intraobserver variability was not researched in this study, but previously found to be good in several studies in less experienced observers.^18^, ^38^

Acknowledging these limitations, we believe that this explorative study provides a relevant novel application of routinely performed, readily available CT imaging for optimization of the identification process of patients undergoing TL at high risk of developing a PCF. More research into the method of quantification of arterial calcification in head and neck cancer patients is warranted and clarification of its relevance for the occurrence of PCF is needed.

## 5 CONCLUSION

Arterial calcification is widespread in patients undergoing laryngectomy and is associated with pharyngocutaneous fistula formation. Extensive arterial calcification on preoperative CT imaging may be taken into consideration as a preoperative risk factor for pharyngocutaneous fistula in patients undergoing laryngectomy.

## Supporting information


**Table S1** presence of arterial calcification and sarcopenia
**Table S2** Sarcopenia and arterial calcification as predictors of pharyngocutaneous fistula

## Data Availability

The data that support the findings of this study are available from the corresponding author upon reasonable request.
