# Supplementary material for: Arterial calcification on preoperative computed tomography imaging as a risk factor for pharyngocutaneous fistula formation after total laryngectomy
Source: Head Neck. 2021 Nov 10;44(2):307–16. doi: 10.1002/hed.26927 (PMC9299602; doi:10.1002/hed.26927)
Supplement: Supplementary file 1 — Table S1 presence of arterial calcification and sarcopenia Table S2 Sarcopenia and arterial calcification as predictors of pharyngocutaneous fistula [file HED-44-307-s001.docx]

Supplementary Data

Table 1: presence of arterial calcification and sarcopenia

| Anatomical location of arterial calcification | Score^a^ | Sarcopenia  (n = 103) | No sarcopenia  (n = 121) | P value |
| --- | --- | --- | --- | --- |
| Ascending aorta | 0  1 | 60  43 | 72  49 | 0.85^b^ |
| Aortic arch | 0  1 | 32  71 | 44  77 | 0.40^b^ |
| Descending aorta | 0  1 | 36  67 | 69  52 | < 0.01^b^ |
| Origins of the brachiocephalic arteries | 0  1 | 28  75 | 39  82 | 0.41^b^ |
| Left extracranial carotid artery | 0  1 | 28  75 | 34  87 | 0.88^b^ |
| Right extracranial carotid artery | 0  1 | 35  68 | 35  86 | 0.42^b^ |
| Left vertebral artery | 0  1 | 87  16 | 107  14 | 0.39^b^ |
| Right vertebral artery | 0  1 | 90  13 | 108  13 | 0.66^b^ |
| Left carotid siphon | 0  1 | 30  73 | 39  82 | 0.62^b^ |
| Right carotid siphon | 0  1 | 33  70 | 42  79 | 0.67^b^ |
| Total arterial calcification score^d^ | Median  IQR | 6.0  4.0 - 8.0 | 5.03.5 - 7.0 | 0.18^e^ |
| Adapted arterial calcification score | Median  IQR | 6.0  3.0 - 7.0 | 5.0  3.0 - 7.0 | 0.19^e^ |

Numbers in bold: significant at the level of p ≤ 0.05

^a^ Score: 0 - none to mild; 1 - moderate to severe

^b^ Pearson Chi square test

^c^ Continuous; score between 0 and 10

^d^ Continuous; score between 0 and 8

^e^ Mann-Whitney U test

Table 2: Sarcopenia and arterial calcification as predictors of pharyngocutaneous fistula

|  | Value | Unadjusted OR^c^ (95% CI) | P value | Adjusted OR^d^ (95% CI) | P value |
| --- | --- | --- | --- | --- | --- |
| Total arterial calcification score | Cont.^a^ | 1.11 (1.00 - 1.27) | 0.04 | 1.12 (1.00 - 1.26) | 0.06 |
| Adapted arterial calcification score | Cont.^b, c^ | 1.19 (1.04 - 1.36) | 0.01 | 1.18 (1.03 - 1.36) | 0.02 |
| Sarcopenia | No  Yes | Ref  1.96 (1.9 - 3.55) | 0.03 | Ref  1.86 (1.02 - 3.39) | 0.04 |

Numbers in bold: significant at the level of p ≤ 0.05

^a^ Continuous; score between 0 and 10

^b^ Continuous; score between 0 and 8

^c^ Entered in multivariable logistic regression analysis instead of the total arterial calcification score

^d^ Univariable logistic regression analysis

^e^ Multivariable logistic regression analysis
